# MCL1 inhibition is effective against a subset of small-cell lung cancer with high MCL1 and low BCL-X_L_ expression

**DOI:** 10.1038/s41419-020-2379-2

**Published:** 2020-03-09

**Authors:** Yuto Yasuda, Hiroaki Ozasa, Young Hak Kim, Masatoshi Yamazoe, Hitomi Ajimizu, Tomoko Yamamoto Funazo, Takashi Nomizo, Takahiro Tsuji, Hironori Yoshida, Yuichi Sakamori, Naoki Nakajima, Toshi Menju, Akihiko Yoshizawa, Hiroshi Date, Toyohiro Hirai

**Affiliations:** 1 Department of Respiratory Medicine, Kyoto University Graduate School of Medicine, Kyoto, Japan; 2 Department of Respiratory Medicine, Takatsuki Red Cross Hospital, Takatsuki, Japan; 3 Department of Diagnostic Pathology, Kyoto University Hospital, Kyoto, Japan; 4 Department of Thoracic Surgery, Kyoto University Graduate School of Medicine, Kyoto, Japan

**Keywords:** Small-cell lung cancer, Apoptosis

## Abstract

There have been few advances in the treatment of small-cell lung cancer (SCLC) because of the lack of targets. MCL1, a member of the anti-apoptotic BCL-2 family, may be a treatment target in several cancers, including SCLC. However, whether the expression profile of the anti-apoptotic BCL-2 family affects MCL1 inhibition strategy is unknown. A tissue microarray (TMA) was created from consecutive patients who were diagnosed with SCLC and had previously undergone surgery at Kyoto University Hospital (Kyoto, Japan) between 2001 and 2017. We used S63845, a MCL1 inhibitor, to assess the cytotoxic capacity in SCLC cell lines including a patient-derived cell line in vitro and in vivo. The combination of S63845 with navitoclax, a double BCL-X_L_/BCL-2 inhibitor, was also employed to examine the comprehensive inhibition of the anti-apoptotic BCL-2 family. Immunohistochemistry of a TMA from patients with surgically resected SCLC demonstrated high MCL1 expression with low BCL-X_L_ and BCL-2 to be the most common expression profile. S63845 was effective in high MCL1- and low BCL-X_L_-expressing SCLC cell lines. S63845 induced BAK-dependent apoptosis in vitro, and the anti-tumor efficacy was confirmed in an in vivo model. Although knockdown of BCL-X_L_ and BCL-2 improved the cytotoxic activity of S63845 and its combination with navitoclax increased the anti-tumor cytotoxicity, the therapeutic range of S63845 with navitoclax was narrow in in vivo studies. Our study suggests MCL1 inhibition therapy be applied for high MCL1- and low BCL-X_L_-expressing SCLC patients.

## Introduction

New cancer drugs against small-cell lung cancer (SCLC) have been awaited because the 3-year survival of extensive-disease SCLC (ED-SCLC) is below 5%^1^. The standard first-line treatment for ED-SCLC is platinum chemotherapy using etoposide or irinotecan^2^. Recently, the addition of atezolizumab, an anti PD-L1 antibody, to standard chemotherapy improved the overall survival and progression-free survival^3^. However, the reported median overall survival is still as short as 12.3 months, and new treatment strategies are needed.

*TP53* is a well-known tumor suppressor gene and is essential for the development of SCLC-like tumors in genetically engineered mouse models and human embryonic stem cell-based models^4,5^. The p53 protein directly upregulates the expression of *PUMA, NOXA, BID*, and *BAX*, which is followed by mitochondrial apoptosis^6–9^. Loss of function of *TP53* due to mutation alters the expression profile of the BCL-2 family, promoting the anti-apoptotic process^10^.

The BCL-2 family is well known for its anti-apoptotic roles in cancer. Members of the BCL-2 family are grouped into three functional subsets: (1) anti-apoptotic proteins (MCL-1, BCL-X_L_, BCL-2, BFL-1/A1, BCL-w, and BCL-b), (2) pro-apoptotic proteins (BAK and BAX), and (3) pro-apoptotic BH3-only motif proteins (NOXA, PUMA, BIM, BID, BIK, BAD, HRK, and BMF)^11^. A previous report suggested that MCL1, BCL-X_L_, and BCL-2 are differentially expressed in SCLC^12^. Thus, targeting of BCL-2 or BCL-X_L_ has been investigated as a possible treatment for SCLC^13,14^, but it is unclear whether direct inhibition of MCL1 is also useful.

MCL1 was first isolated from human myeloid leukemia cells^15^. Interacting with BAK, MCL1 has opposing effects on mitochondrial apoptosis^16^. The MCL1 protein is widely expressed in normal tissues and its gene amplification is frequently found in various cancers^17,18^. MCL1 plays a role in maintaining the viability of acute myeloid leukemia, multiple myeloma, hepatocellular carcinoma, prostate cancer, and non-SCLC^19–22^. Although high MCL1 expression was often observed in several studies using SCLC cell lines^12,13,23^, the expression profile of MCL1 in SCLC patients remains poorly investigated.

S63845 is the first MCL1 inhibitor with cytotoxic cellular potency^24^. It is effective against hematological malignancies, breast cancer, nasopharyngeal cancer, and prostate cancer^24–27^. Until S68345 was developed, selective direct and potent inhibition of MCL1 was difficult because of the high affinity of the BH3 pocket to other binding partner proteins, such as BAK, BAX, and BIM. Previously, many different strategies were used to indirectly inhibit MCL1 to treat cancer. Alvocidib, a cyclin-dependent kinase (CDK) 9 inhibitor, downregulates MCL1, resulting in anti-tumor effects on hematological malignancies^28^. Roniciclib, a pan-CDK inhibitor, reduces MCL1 expression^29^, but a phase 2 study of roniciclib in combination with cytotoxic chemotherapy did not improve the progression-free survival of patients with ED-SCLC^30^. A phase 2 study of sorafenib, which also reduces MCL1 expression, in conjunction with chemotherapy demonstrated significant toxicity and poor overall median survival^27,31^. Thus, indirect inhibition of MCL1 for SCLC treatment was considered difficult because of its dose-limiting toxicity.

Therefore, the direct inhibition of MCL1 may be effective. Recently, S63845 was reported to be effective in MYC paralog-amplified human SCLC cell lines^32^. Based on the aforementioned evidence and focusing on the anti-apoptotic BCL-2 family, we examined the anti-apoptotic BCL-2 family expression profile in SCLC patients and S63845 efficacy in SCLC cell lines.

## Materials and methods

### Patient selection

Patients who were diagnosed with SCLC and had previously undergone surgery at Kyoto University Hospital (Kyoto, Japan) between 2001 and 2017 were enrolled. The relevant clinical data were collected by retrospective review of their charts. Expert pathologists (N.N. and A.Y.) reviewed and confirmed the pathological diagnosis of consecutive SCLC to create a tissue microarray (TMA) block containing 28 cores. Twenty-five of the 28 cores were linked to clinical data. The study protocol had been prepared in accordance with the Declaration of Helsinki and was approved by the Kyoto University Graduate School and Faculty of Medicine Ethics Committee (R0854-2). All patients provided written informed consent for clinical investigation.

### Immunohistochemistry and imaging analysis

A TMA was sectioned at a thickness of 4 µm, and the sections were deparaffinized for immunohistochemistry. Immunohistochemical stainings for MCL1, BCL-X_L_, and BCL-2 were performed by the conventional avidin–biotin-peroxidase complex (ABC) method. Incubation and washing procedures were carried out at room temperature if they were not specified. After deparaffinization and antigen retrieval by microwaving in ethylenediaminetetraacetic acid (EDTA) buffer for 20 min, endogenous peroxidase activity was blocked by 0.3% H_2_O_2_ in methyl alcohol for 30 min. Subsequently, the primary antibody was applied overnight at 4 °C. The concentrations of primary antibodies are listed in Supplementary Table 1. The sections were next incubated with a biotinylated secondary antibody diluted 1:300 in PBS for 40 min, followed by washing with PBS (six times, 5 min). ABC (ABC-Elite, Vector Laboratories, Burlingame, CA) at a dilution of 1:100 in bovine serum albumin (BSA) was applied for 50 min. After washing with PBS (six times, 5 min), coloring reaction was carried out with DAB and nuclei were counterstained with hematoxylin. Immunohistochemical stainings for achaete-scute homolog 1 (ASCL1), neurogenic differentiation factor 1 (NEUROD1), yes-associated protein 1 (YAP1), and POU class 2 homeobox 3 (POU2F3) were performed by Ventana Discovery Ultra (Roche, Tucson, AZ). For antigen retrieval, cell conditioning 1 (CC1) buffer (Roche, Tucson, AZ) was used for 32 min. Primary antibodies were applied to TMA sections for 32 min (Supplementary Table 1). Antigen–antibody reactions were observed using Ventana UltraView Universal DAB Detection kit (Roche, Tucson, AZ).

All stained slides were digitalized using a NanoZoomer S360 digital slide scanner (Hamamatsu Photonics, Hamamatsu, Japan). Annotations and analysis were conducted using HALO image analysis software with the CytoNuclear IHC v1.6 algorithm module (Indica Labs, NM) while consulting a pathological expert (A.Y.). Tumor cells were classified by the HALO tissue classifier (random forest). The positivity threshold for staining was determined empirically based on controls and the intensity was classified from 0 to 3. The H-score was calculated automatically according to HALO configuration settings (Supplementary Table 2). When the H-score was 150 or greater, it was defined as high expression. Positive staining was defined as an H-score of 100 or more for ASCL1, NEUROD1, YAP1, and POU2F3. The tumor proportion score (TPS) was also calculated.

### Cell lines and cell culture

The SCLC cell lines DMS-114, DMS-53, SW1271, and NCI-H69 were purchased from the American Type Culture Collection (Manassas, VA). KTOR201 was established from pleural fluid of a clinically and pathologically diagnosed SCLC patient. PC-9 (epidermal growth factor receptor: EGFR Ex19 del) was purchased from European Collection of Cell Cultures (a Culture Collection of Public Health England, Porton Down, England). All cell lines were cultured and maintained in RPMI 1640 medium (Nacalai Tesque, Kyoto, Japan) supplemented with 8% heat-inactivated FBS (Signa-Aldrich, St. Louis, MO) and 1% Penicillin/Streptomycin (Gibco™, Thermo Fisher Scientific, Waltham, MA) at 37.0 °C in 5% CO_2_. Cell lines were routinely tested for mycoplasma by MycoAlert™ (Lonza, Basel, Switzerland).

### Establishment of primary SCLC cell lines

Pleural effusion from a patient with SCLC was collected by 16-G needle aspiration. Two hundred millliliters of pleural effusion was centrifuged at 160 × *g* for 5 min at room temperature to pellet the cells. The cells were added to 2.5 ml of phosphate buffer saline (PBS) and 5 ml of red blood cell lysis buffer (Roche Diagnostics, Mannheim, Germany), and the solution was incubated for 6 min at room temperature. The solution was then centrifuged at 160 × *g* for 5 min at room temperature and the pellet was cultured in a Petri dish in RPMI and 8% FBS medium.

### Drugs

S63845, navitoclax, Z-VAD-FMK, cisplatin, and etoposide for the cell culture and in vivo experiments were purchased from Active Biochem (Kowloon, Hong Kong), ChemScene (Monmouth Junction, NJ), Selleck Chemicals (Houston, TX), Wako (Kyoto, Japan), and LC laboratories (Woburn, MA), respectively. For the in vitro study, they were dissolved in dimethyl sulfoxide (DMSO) (Nacalai Tesque, Kyoto, Japan) at a concentration of 5 mmol/L. DMSO was also used as a vehicle control in the in vitro study.

### Cell viability and drug sensitivity assays

This assay was performed as described previously^33^. Cells (5000 cells/well) were cultured in 96-well plates and incubated with stepwise concentrations of the indicated drugs or vehicle control medium for 72 h. Viable cells were quantified using the CellTiter-Glo 2.0 Luminescent Cell Viability Assay (Promega, Madison, WI). Luminescence was measured by ARVO X3 (PerkinElmer, Waltham, MA). Half maximal (50%) inhibitory concentration (IC50) values were calculated using a non-linear regression model with a sigmoidal dose response by GraphPad Prism 8.1 (GraphPad software, La Jolla, CA).

### Apoptosis assay

Cells (1250 cells/well) were cultured in 386-well plates and incubated with DMSO, 100 or 200 nM S63845 for 6 h. The activity of caspase 3/7, 8, and 9 was assessed using the Caspase-Glo 3/7 Assay, Caspase-Glo 8 Assay, and Caspase-Glo 9 Assay (Promega, Madison, WI), respectively, according to the manufacturer’s recommendations. The measured value was corrected by the cell number using the CellTiter-Glo 2.0 Luminescent Cell Viability Assay.

The FITC Annexin V Apoptosis Detection Kit 1 (BD biosciences, San Jose, CA) was used to detect apoptosis. One million cells were suspended in 2 ml of culture medium and incubated at 37.0 °C in 5% CO_2_ for 4 h after treatment with DMSO or 100 nM S63845 in the presence or absence of 50 µM Z-VAD-FMK. According to the manufacturer’s protocol, the cells were stained with FITC-conjugated annexin V and PI, followed by flow cytometric analysis using BD FACSMelody™ (BD biosciences, San Jose, CA). The percentage of the FITC annexin V-positive population was defined as the percentage of apoptosis.

### Immunoblotting

SDS–PAGE and immunoblotting were performed as described previously^34^. Information of primary antibodies was summarized in the Supplementary Table 1. Secondary antibodies were purchased from Cell Signaling Technology (Danvers, MA). The primary and secondary (1:4000) antibodies were diluted in 2.5% BSA (Nacalai Tesque, Kyoto, Japan)/tris-buffered saline with tween 20 (TBS-T).

### Co-immunoprecipitation (Co-IP)

Growing SCLC cells were incubated with DMSO or S63845 for 4 h. Cell lysates were prepared with a solution of NP40 lysis buffer (50 mM Tris with pH 8.4, 1 mM EDTA, 150 mM NaCl, 1 mM DTT, 0.5% (v/v) NP40, PhosSTOP™ (Sigma-Aldrich, St. Louis, MO), and cOmplete mini™ (Roche)). The pellet was centrifuged at 15,000 × *g* for 10 min at 4 °C, and the collected supernatant was uniformly adjusted in concentration for Co-IP.

Co-IP was performed using the Dynabeads™ Protein G Immunoprecipitation Kit (Invitrogen, Waltham, MA) according to the manufacturer’s instructions. The rabbit IgG isotype (Cell Signaling Technology, Danvers, MA) and the MCL1 antibody (Abcam, Cambridge, UK) were diluted 1:200. After elution by NP40 lysis buffer, SDS sample buffer (the same as for immunoblotting) was added and heated for 5 min at 95 °C. The immunoprecipitants were subjected to immunoblotting. In order to reduce the detection of the denatured rabbit IgG light and heavy chains of the primary antibody used for Co-IP, mouse anti-rabbit IgG (Conformational specific) (1:2000) (Cell Signaling Technology, Danvers, MA) was used before the secondary antibody reaction.

### Transfection of small interfering RNA (siRNA)

siRNA oligonucleotides for *MCL1, BCL-X*_*L*_*, BCL-2, BAK*, and *BAX* were purchased from Thermo Fisher Scientific (Stealth RNAi™ or Silencer^®^ Select siRNA) (Supplementary Table 3). Cells were transfected with siRNA oligonucleotides at a final RNA concentration of 20 nM using Lipofectamine^®^ RNAiMAX Transfection Reagent (Invitrogen, Waltham, MA). A reverse transfection method was employed following the manufacturer’s instructions. Seventy-two hours after transfection, total protein was extracted for immunoblotting. In the cell viability assay, cells (4000 cells/well) in 96-well plates were transfected and frozen at 0 or 72 h after transfection (−80 °C). After freezing, both plates were thawed simultaneously, and viable cell numbers were quantified using CellTiter-Glo as described above. The relative cell number ratio was calculated with the formula: 72 h/0 h. The graph was expressed as % of control.

### Quantitative real-time polymerase chain reaction (qRT-PCR)

Total RNA was extracted from cultured cells using the PureLink^®^ RNA mini kit (Thermo Fisher Scientific, Waltham, MA). Gene expression was measured by qRT-PCR assay with each reaction containing 100 ng of total RNA, One Step SYBR^®^ PrimeScript™ RT-PCR Kit II (Takara Bio Inc, Shiga, Japan), and a primer pair designed to amplify the target mRNA (Supplementary Table 4). Reactions were run on a QuantStudio™ 3 Real-Time polymerase chain reaction system (Applied Biosystems, Foster City, CA) for quantitation.

### Xenograft models

Male SCID beige (CB17.Cg-PrkdcscidLystbg-J/CrlCrlj) mice were purchased at 5–6 weeks of age from Charles River Laboratories, Japan (Yokohama, Japan). They were subcutaneously inoculated into the back with 100 µl (50:50 mixture of Matrigel^®^ (Corning, Corning, NY) and growth medium) containing 5.0 × 10^6^ of DMS114 or 3.0 × 10^6^ of DMS53 or 1.5 × 10^6^ of KTOR201 cells. The width and length of the tumors were measured at least two times a week using electronic calipers. The tumor volume was calculated using the formula: (length × width^2^)*0.51. When the tumor volume exceeded 150 mm^3^, mice were randomized into treatment or vehicle groups. S63845 was formulated in 25 mM HCl and 20% 2-hydroxy propyl β-cyclo dextrin (Wako), and administered intravenously at the dose of 25 mg/kg on day 0 and day 3. Cisplatin and etoposide were formulated in 1% DMSO and saline. Cisplatin was administered intraperitoneally at the dose of 3 mg/kg on day 0. Etoposide was administered intraperitoneally at the dose of 4 mg/kg on days 0–2. Navitoclax was formulated in 60% Phosal 50 PG (Thermo Fisher Scientific, Waltham, MA), 30% polyethylene glycol 400 (Wako), and 10% ethanol (Wako), and administered orally on days 0–4.

All animal experiments and research plans were approved by the Animal Research Committee of Kyoto University (ID: MedKyo 18298/19295) and were conducted in accordance with ARRIVE guidelines.

### Statistics

Continuous variable data were expressed as mean ± SEM. *p*-Values of all combinations were calculated using one-way ANOVA for single variables or two-way ANOVA for more than two variables with Tukey’s correction or Sidak’s correction for multiple comparisons. *p*-Values of <0.05 were defined as significant. All statistical analyses were performed and visualized by GraphPad Prism 8.1 (GraphPad Software, San Diego, CA). All in vitro experiments were independently confirmed three times.

## Results

### High expression of MCL1 with low expression of BCL-X_L_ and BCL-2 is most common in SCLC

**Fig. 1:**
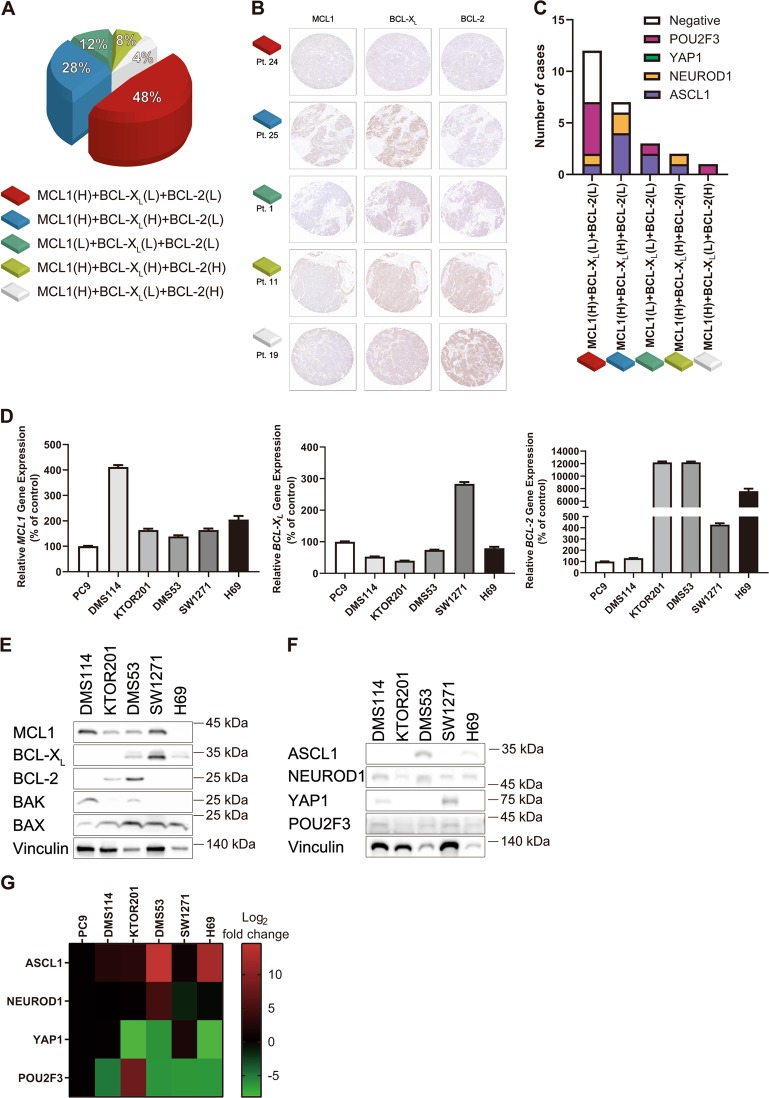
High expression of MCL1 with low expression of BCL-X_L_ and BCL-2 is the most common profile in SCLC. **a** An H-score of >150 points was defined as high. The expression profiles of MCL1, BCL-X_L_, and BCL-2 in each case are shown as a percentage of all 25 cases. (H) or (L) means high or low expression, respectively. **b** Representative images of immunohistochemical expression of MCL1, BCL-X_L_, and BCL-2 in TMA from SCLC patients. The patient numbers shown in the figure match the patient numbers shown in Table 1. **c** Bar graph indicating the number of cases of ASCL1, NEUROD1, YAP1, and POU2F3 subtypes according to the expression profiles of MCL1, BCL-X_L_, and BCL-2. **d** Relative gene expression of *MCL1*, *BCL-X*_*L*_, and *BCL-2* normalized to *GAPDH* in SCLC cell lines by qRT-PCR. Results are expressed as mean ± SEM (*N* = 4). **e** Expression of MCL1, BCL-X_L_, BCL-2, BAK, and BAX in SCLC cell lines was measured by immunoblotting. **f** Expression of ASCL1, NEUROD1, YAP1, and POU2F3 in SCLC cell lines was measured by immunoblotting. **g** Gene expression analysis heat map of *ASCL1*, *NEUROD1*, *YAP1*, and *POU2F3* normalized to *GAPDH* in SCLC cell lines. Red, high expression; green, low expression based on the expression level of PC9.

We first performed immunohistochemistry using a TMA slide consisting of surgically resected SCLC. Twenty-five cores were stained by MCL1, BCL-X_L_, and BCL-2, and the H-score and TPS were uniformly evaluated by HALO™. MCL1^high^/BCL-X_L_^low^/BCL-2^low^ population was observed in 12 of 25 cases (Fig. 1a, b). Recently, Rudin et al. proposed four SCLC molecular subtypes based on the expression of ASCL1, NEUROD1, YAP1, or POU2F3^35^. We assessed the expression profile of these four transcription factors in the TMA (Supplementary Fig. 1A). All patient characteristics and immunohistochemical expression profiles are listed in Table 1. When positive staining was defined as an H-score of 100 or more, cases could not be clearly classified into a particular subtype. Six cases showed multiple positives and six cases showed all negatives. The NEUROD1 subtype sometimes overexpresses ASCL1^36^, and the POU2F3 and YAP1 subtypes express neither ASCL1 nor NEUROD1^37,38^. Based on these reports, we classified positive expression of ASCL1/NEUROD1 as the NEUROD1 subtype, positive expression of ASCL1/POU2F3 as the ASCL1 subtype, positive expression of ASCL1/NEUROD1/POU2F3 as the NEUROD1 subtype, and positive expression of NEUROD1/YAP1/POU2F3 as the NEUROD1 subtype. The MCL1^high^/BCL-X_L_^low^/BCL-2^low^ population mostly belonged to the POU2F3 subtype and the POU2F3 subtype was not observed in the BCL-X_L_^high^ population (Fig. 1c).

**Table 1:** Patient characteristics, and immunohistochemistry scores of MCL1, BCL-X_L_, BCL-2, ASCL1, NEUROD1, YAP1, and POU2F3.

Patient number	Age at diagnosis	Sex	Smoking history (pack year)	TNM stage	H-scores (0–300)							Tumor proportion scores (%)		
					MCL1	BCL-X_L_	BCL-2	ASCL1	NEUROD1	YAP1	POU2F3	MCL1	BCL-X_L_	BCL-2
1	64	M	45	pT4N1M0	127.80	57.44	9.39	41.22	22.29	0.66	125.73	29.6	3.6	0.2
2	67	F	58.75	pT1aN0M0	144.07	119.87	31.93	228.87	0.05	7.02	37.99	39.1	27.1	2.0
3	74	F	50	pT3N1M0	234.44	154.43	48.74	255.36	202.52	2.62	13.79	93.3	52.8	2.6
4	70	M	50	pT1N1M0	209.41	204.52	51.01	212.49	145.21	4.33	22.36	84.5	78.4	2.6
5	45	M	30	pT3N1M0	200.00	125.44	0.08	0.00	1.12	6.45	16.05	86.9	32.7	0.0
6	65	M	45	pT3N0M0	213.52	113.61	7.66	36.79	0.21	5.63	100.41	86.4	32.0	0.5
7	68	M	NA	pTXNXM1	230.12	151.69	117.09	135.78	61.22	25.41	63.18	93.6	50.5	35.8
8	74	F	23	pT1aN0M0	221.33	64.68	107.08	34.59	4.54	3.65	29.15	90.7	4.4	25.1
9	51	M	60	pT2N1M0	233.89	147.46	124.14	215.73	5.93	14.25	87.95	93.1	47.4	35.7
10	71	M	45	pT2aN0M0	234.79	178.09	32.44	100.74	0.38	3.85	176.43	97.2	65.6	2.5
11	71	F	0	pT2aN2M0	216.02	159.07	157.64	117.08	5.05	43.43	66.86	93.2	54.1	54.3
12	87	M	60	pT2aN0M0	222.88	176.25	96.24	23.39	1.80	1.39	88.79	88.9	63.2	23.5
13	61	M	34	pT3N0M0	252.90	118.12	4.41	63.76	42.88	3.67	185.68	96.5	25.5	0.1
14	62	F	42	pT1aN0M0	207.50	141.07	3.00	0.15	26.41	85.48	196.88	87.4	42.4	0.2
15	72	M	67.5	pT3N0M0	171.11	9.56	0.00	0.040	2.37	4.22	158.13	61.4	0.1	0.0
16	70	M	90	pT2aN0M0	282.23	195.83	218.90	240.22	171.37	60.73	128.52	99.0	74.7	75.6
17	79	M	45	pT1bN0M0	170.21	81.09	0.11	0.35	0.01	1.25	54.13	66.5	8.1	0.0
18	59	M	80	pT1aN0M0	224.04	72.22	15.75	87.11	24.48	25.33	91.73	92.8	6.8	0.8
19	81	F	45	pT1aN0M0	219.89	110.47	221.06	0.02	10.96	9.75	107.39	88.4	22.4	78.4
20	74	F	0	pT1cN1M0	218.4	188.02	22.44	174.60	3.57	8.23	68.91	87.8	68.6	2.8
21	53	M	33	pT1N1M0	148.62	144.69	2.04	115.74	13.18	63.72	102.61	41.4	45.8	0.1
22	72	F	50	pT3aN0M0	242.82	93.47	10.72	17.55	233.10	159.19	138.08	87.8	18.7	0.5
23	79	M	60	pT1bN0M0	251.67	79.58	84.18	98.43	0.43	27.02	159.21	95.1	13.0	19.0
24	74	F	24	pT2aN0M0	188.44	5.34	7.43	15.52	22.07	24.66	70.65	75.5	0.0	0.2
25	73	M	47	pT2aN0M0	273.66	211.42	10.19	195.58	0.36	26.31	83.02	93.7	76.9	0.8

We next investigated the anti-apoptotic BCL-2 family profile in SCLC cell lines. We established a primary SCLC cell line, KTOR201, from pleural effusion of a female patient with ED-SCLC after progression on cisplatin and etoposide. To confirm whether KTOR201 had the characteristics of SCLC, mRNA expression of the neural cell adhesion molecule, chromogranin A, and synaptophysin was evaluated. These neuroendocrine markers of KTOR201 were expressed significantly higher than in PC9 (an adenocarcinoma cell line), like other SCLC cell lines (Supplementary Fig. 1B). The expression of MCL1, BCL-X_L_, and BCL-2 in five SCLC cell lines (DMS114, KTOR201, DMS53, SW1271, and H69) was evaluated (Fig. 1d, e). MCL1 protein was highly expressed in DMS114, KTOR201, DMS53, and SW1271, whereas BCL-X_L_ protein was highly expressed in DMS53, SW1271, and H69, and BCL-2 protein was highly expressed in KTOR201 and DMS53. Expression profiles of MCL1, BCL-X_L_, and BCL-2 showed inconsistencies between mRNA and protein data. The subtypes of these five SCLC cell lines were classified by immunoblotting and qRT-PCR (Fig. 1f, g). As in the study by Rudin et al., DMS114 and SW1271 highly expressed YAP1, and DMS53 and H69 highly expressed ASCL1. KTOR201 showed high expression of *POU2F3* at the mRNA level and was considered to belong to the POU2F3 subtype.

### S63845 is effective in high MCL1- and low BCL-X_L_-expressing SCLC cell lines in vitro and in vivo

**Fig. 2:**
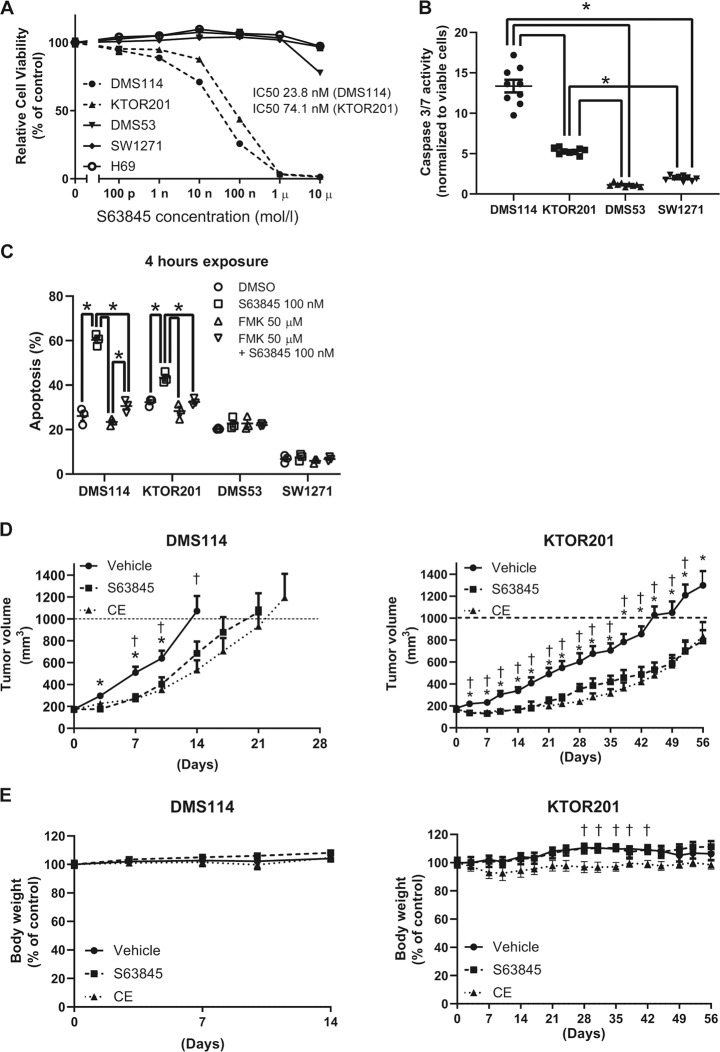
S63845 is effective against SCLC with high MCL1 and low BCL-X_L_ expression in vitro and in vivo. **a** Growth inhibition of SCLC cell lines treated with S63845 assessed by the CellTiter-Glo assay. Results are expressed as mean ± SEM (*N* = 5). IC50 values were calculated using the growth inhibition assay. **b** Caspase 3/7 activity in DMS114 and KTRO201 treated with 100 nM S63845 for 6 h was significantly higher than that in SW1271 and DMS53. Data are presented as fold-change in activity relative to vehicle as means ± SEM (*N* = 9). One-way ANOVA with Tukey’s multiple comparison test was used. *p < 0.05. **c** The annexin V-positive population was defined as apoptotic. The percentages of apoptosis in SCLC cell lines were plotted after 4 h of exposure to DMSO, 100 nM S63845, 50 µM Z-VAD-FMK, or both inhibitors. Results are expressed as mean ± SEM (*N* = 3). The difference in the percentage of apoptosis in each SCLC cell line among the four groups was assessed by two-way ANOVA with Tukey’s multiple comparison test. *p < 0.05. **d** Tumor volume in SCID beige mice bearing DMS114 or KTOR201 after treatment with vehicle control (*N* = 14, DMS114; *N* = 8, KTOR201), cisplatin/etoposide (CE) (*N* = 7, DMS114; *N* = 8, KTOR201), S63845 (*N* = 14, DMS114; *N* = 7, KTOR201). Results are expressed as mean ± SEM. Two-way ANOVA with Tukey’s multiple comparison test demonstrated a significant difference between vehicle and S63845 (*) and between vehicle and CE (†). *, †*p* < 0.05. **e** Percent body weight change of SCID beige mice bearing DMS114 and KTOR201 during treatment with indicated agents. Results are expressed as mean ± SEM. Two-way ANOVA with Tukey’s multiple comparison test demonstrated a significant difference between vehicle and CE (†). †*p* < 0.05.

We assessed the efficacy of S63845 in the five SCLC cell lines. S63845 reduced cell viability of DMS114 and KTOR201 (Fig. 2a). S63845 causes apoptosis by binding the BH3 site of MCL1^24^. To confirm the induction of apoptosis by S63845, caspase 3/7 activity was evaluated. Caspase 3/7 activity was increased by S63485 exposure in DMS114 and KTOR201 (Fig. 2b). Since caspase 3/7 activity in KTOR201 after treatment with 100 nM S63845 was lower than that in DMS114, the activities of other caspases were also assessed. While caspase 8 and 9 activity levels in KTOR201 were also lower than those in DMS114 after treatment with 100 nM S63845 (Supplementary Fig. 2A), the activities of caspase 3/7, 8, and 9 in KTOR201 were increased after treatment with 200 nM S63845 (Supplementary Fig. 2B). The high annexin V population in DMS114 and KTOR201 was due to S63485 exposure, and the annexin V population reduced to the control level when a pan-caspase inhibitor (Z-VAD-FMK) was introduced before exposure to S63845 (Fig. 2c, Supplementary Fig. 3A). This suggests that S63845 causes caspase-dependent cell death.

A xenograft model was used to evaluate the anti-tumor activity in vivo. DMS114 or KTOR201 were engrafted to the back of SCID beige mice, and S63845 or cisplatin with etoposide (CE) were, respectively, administered. S63845 or CE significantly reduced the tumor volume compared with the vehicle (Fig. 2d). The anti-tumor efficacy of S63845 was comparable to that of CE. No body weight loss or ulceration was observed in the S63845 group (Fig. 2e).

### S63845 disrupts MCL1:BAK complex to cause apoptosis

**Fig. 3:**
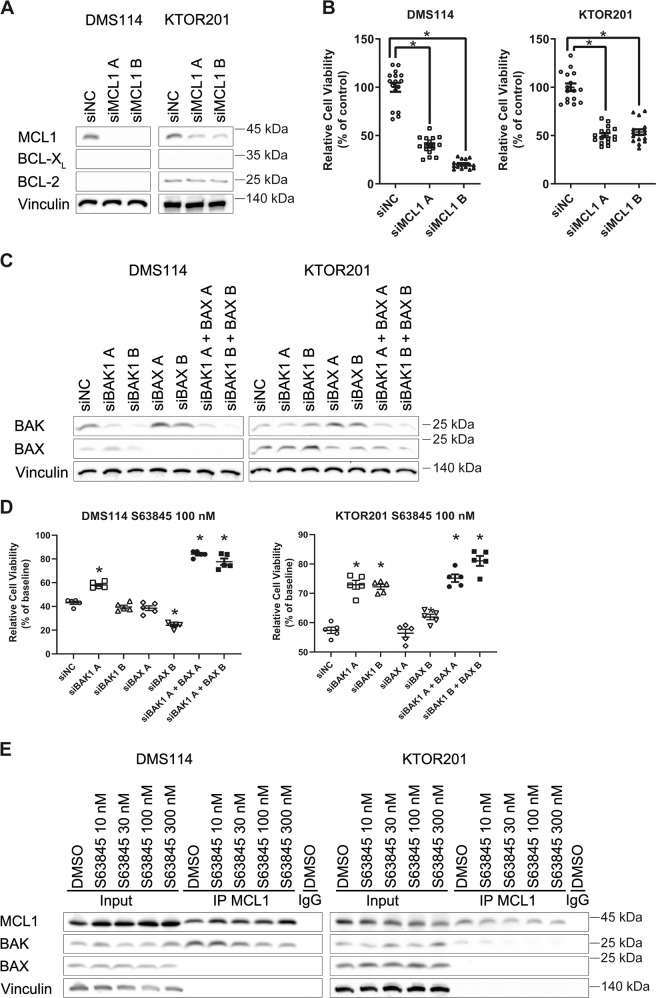
S63845 induces apoptosis by targeting MCL1 to disrupt the MCL1:BAK complex. **a** Immunoblotting analysis of lysates from DMS114 and KTOR201 following siRNA-mediated MCL1 knockdown, which affected MCL1, BCL-X_L_, and BCL-2 expression. **b** Cell growth assays following siRNA-mediated MCL1 knockdown using two different MCL1-directed RNAi sequences (A and B) in DMS114 and KTOR201. Relative cell viability was measured 72 h after transfection with siRNA using CellTiter-Glo. Results are expressed as mean ± SEM (*N* = 16). One-way ANOVA with Tukey’s multiple comparison test was used. *p < 0.05. **c** Immunoblotting analysis of lysates from DMS114 and KTOR201 following siRNA-mediated BAK1, BAX, or double knockdown affecting BAK and BAX expression. **d** Cell growth assays after 72 h of exposure to 100 nM S63845 following siRNA-mediated BAK1, BAX, or double knockdown. Results are expressed as mean ± SEM (*N* = 5). One-way ANOVA with Tukey’s multiple comparison test was used. *p < 0.05 versus siNC group. **e** DMS114 and KTOR201, treated or not-treated with S63845, were subjected to anti-MCL1 immunoprecipitation. The input (100%) and immunoprecipitants were assessed by immunoblot analysis. *p < 0.05.

S63845 is known to be a selective MCL1 inhibitor. To examine whether single knockdown of MCL1 results in anti-tumor activity, siRNA was transfected to DMS114 and KTOR201. As with S63845, the cell viability of DMS114 and KTOR201 was significantly reduced to less than about half compared with the negative control (Fig. 3a, b). S63845 with knockdown of MCL1 decreased the cell viability of DMS114 and KTOR201 but not that of DMS53 and SW1271 (Supplementary Fig. 4A). This suggests that either S63845 has MCL1 selectivity but does not completely inhibit MCL1 at low doses or that DMS114 and KTOR201 may be responding to an off-target effect of S63845.

The BH3 site of MCL1 is known as the binding site for BAK and BAX, which causes apoptosis. Knockdown of both BAK and BAX ameliorated the decrease in cell viability by S63845 (Fig. 3c, d). Co-IP of MCL1 demonstrated that S63845 inhibited binding of BAK to MCL1 in a S63485 concentration-dependent manner (Fig. 3e). Thus, the anti-tumor effects of S63845 were considered to be the induction of apoptosis via MCL1 inhibition.

### High expression of BCL-X_L_ and BCL-2 antagonizes S63845 efficacy

**Fig. 4:**
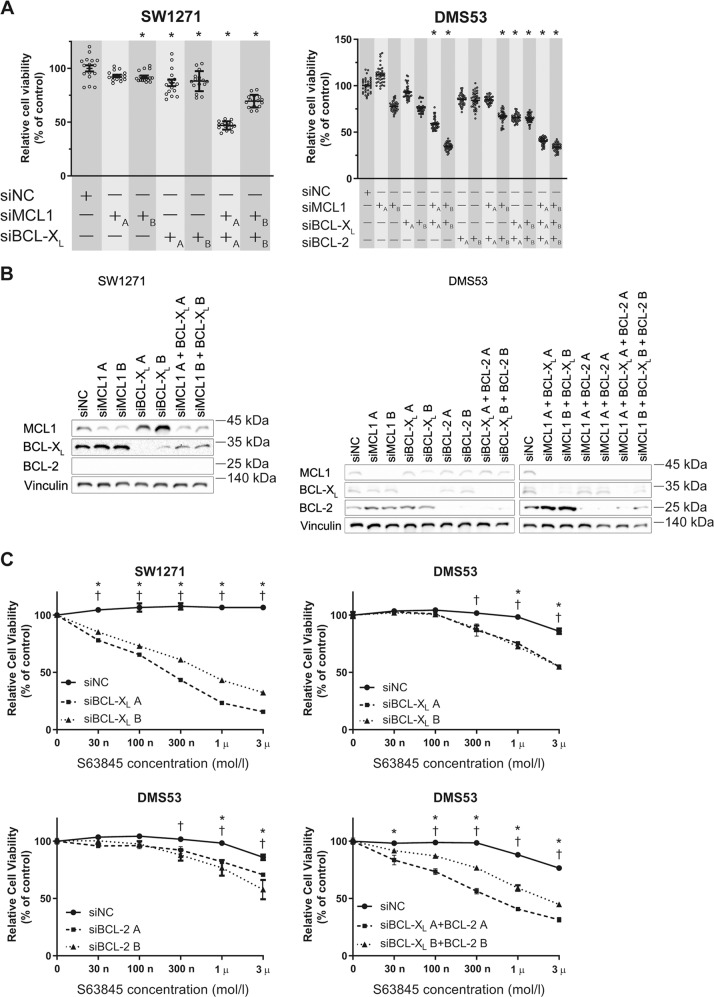
Knockdown of BCL-X_L_ and BCL-2 improves the cytotoxic activity of S63845 in S63845-resistant cell lines. **a** Cell growth assays following siRNA-mediated knockdown of MCL1, BCL-X_L_, or both in SW1271, and that of MCL1, BCL-X_L_, BCL-2, or all in DMS53 using two different RNAi sequences (A and B). Relative cell viability measured 72 h after transfection with siRNA using CellTiter-Glo. Results are expressed as mean ± SEM (*N* = 16, SW1271; *N* = 36, DMS53). One-way ANOVA with Tukey’s multiple comparison test was used. *p < 0.05 versus siNC group. **b** Immunoblotting analysis of lysates from SW1271 and DMS53 following siRNA-mediated knockdown of MCL1, BCL-X_L_, BCL-2, or all affecting MCL1, BCL-X_L_, and BCL-2 expression. **c** Cell viability assay of SW1271 transfected with BCL-X_L_ siRNA or negative control siRNA treated with S63845 72 h after transfection. Cell viability assay of DMS53 transfected with BCL-X_L_, BCL-2, or both siRNA, or negative control siRNA treated with S63845 72 h after transfection. Results are expressed as mean ± SEM (*N* = 5). Two-way ANOVA with Tukey’s multiple comparison test demonstrated a significant difference between siNC and RNAi sequence A (*) and between siNC and RNAi sequence B (†) for each S63845 concentration. *, †*p* < 0.05.

S63845 was not effective in SW1271 and DMS53 regardless of the protein expression of MCL1. In a previous report, high BCL-X_L_ expression was inversely correlated with the sensitivity of S63845^24^. We investigated the influence of combined inhibition of the anti-apoptotic BCL-2 family. Double knockdown of MCL1 and BCL-X_L_ reduced the cell viability of SW1271 and DMS53 (Fig. 4a, b). Double knockdown of MCL1 and BCL-2 also reduced the cell viability of DMS53, and triple knockdown of MCL1, BCL-X_L_, and BCL-2 gave similar results. The sensitivity to S63845 of SW1271 and DMS53 was improved by the knockdown of BCL-X_L_ and BCL-2 (Fig. 4c). On the other hand, the sensitivity to S63845 of KTOR201 was slightly improved by the knockdown of BCL-2 (Supplementary Fig. 4B, C).

**Fig. 5:**
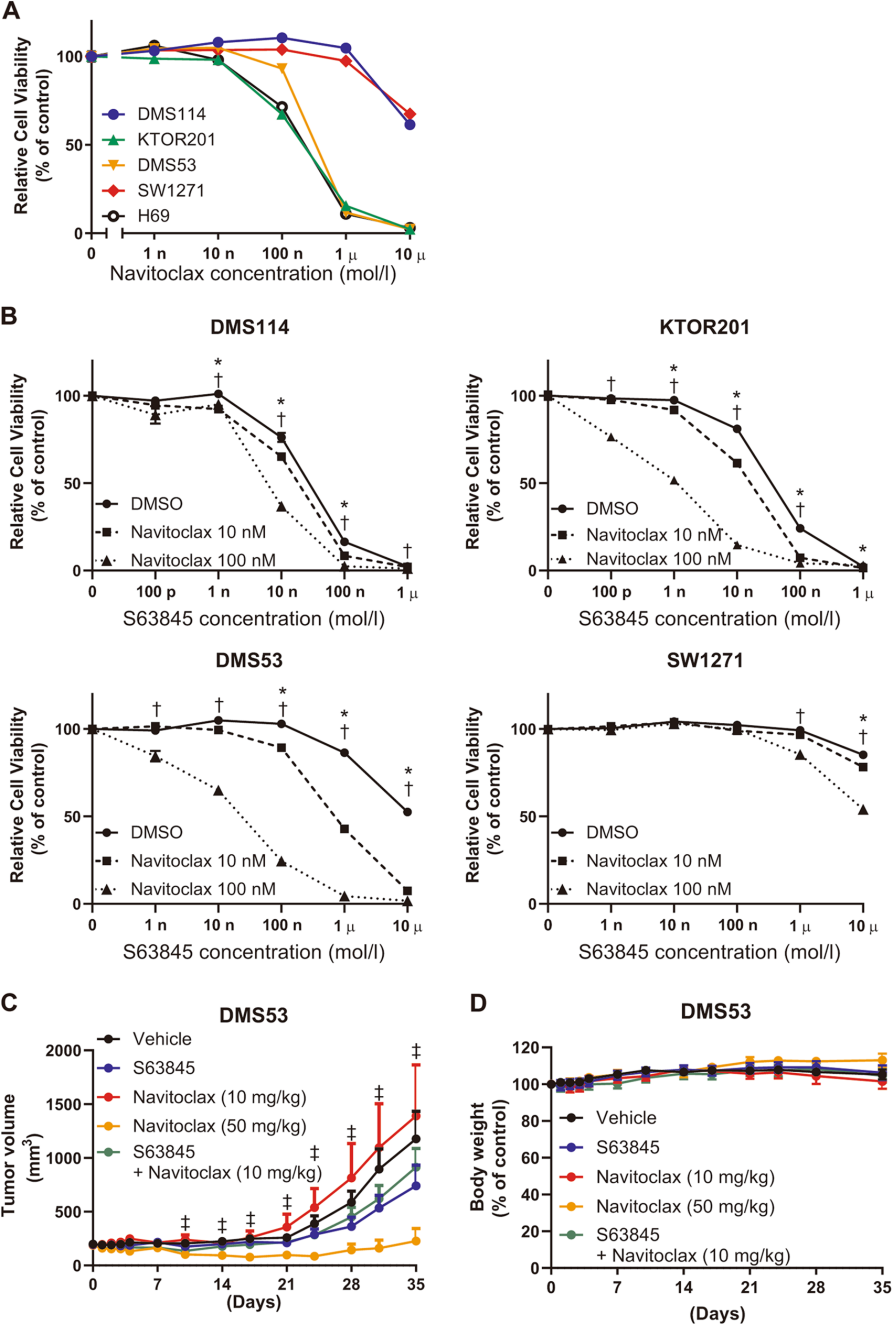
Comprehensive inhibition of MCL1, BCL-X_L_, and BCL-2 is intolerable in vivo. **a** Growth inhibition assessed by the CellTiter-Glo assay of SCLC cell lines treated with navitoclax. Results are expressed as mean ± SEM (*N* = 5). **b** Cell viability assay of DMS114, KTOR201, SW1271, and DMS53 treated with S63845 in combination with navitoclax. Results are expressed as mean ± SEM (*N* = 5). Two-way ANOVA with Tukey’s multiple comparison test demonstrated a significant difference between DMSO and 10 nM navitoclax (*) and between DMSO and 100 nM navitoclax (†) for each S63845 concentration. *,†*p* < 0.05. **c** Tumor volume of SCID beige mice bearing DMS53 after treatment with vehicle control (*N* = 10), S63845 (25 mg/kg, *N* = 10), navitoclax (10 mg/kg, *N* = 7), navitoclax (50 mg/kg, *N* = 6), and S63845 (25 mg/kg) + navitoclax (10 mg/kg, *N* = 10). Results are expressed as mean ± SEM. Two-way ANOVA with Tukey’s multiple comparison test demonstrated a significant difference between vehicle and navitoclax at 50 mg/kg (‡). **d** Percent body weight change of SCID beige mice bearing DMS53 during treatment with indicated agents. Results are expressed as mean ± SEM.

Navitoclax is a dual inhibitor of BCL-X_L_ and BCL-2, and it reduced the cell viability of KTOR201 and DMS53, but not of DMS114 or SW1271 in vitro (Fig. 5a). The combination of S63845 and navitoclax reduced the cell viability of DMS114, KTOR201, DMS53, and SW1271 in a concentration-dependent manner (Fig. 5b). Next, we examined the synergistic effects of S63845 and navitoclax using xenograft model harboring DMS53. In a previous report, 100 mg/kg/day of navitoclax had an objective response rate of 100%^14^. As comprehensive inhibition of the anti-apoptotic BCL-2 family may be toxic, we started at 50 mg/kg/day of navitoclax and 25 mg/kg of S63845 for the in vivo experiment. In the preliminary experiment, 6 of 6 mice were dead after the second administration of S63845 days without weight loss. The dose of 25 mg/kg/day of navitoclax and 25 mg/kg of S63845 also resulted in death. Thus, we administered 10 mg/kg/day of navitoclax and 25 mg/kg of S63845 to SCID beige mice harboring DMS53. Although 50 mg/kg/day of navitoclax reduced the tumor volume, the combination of 10 mg/kg/day of navitoclax with 25 mg/kg of S63845 did not reduce the tumor volume compared with the vehicle (Fig. 5c). No body weight loss or ulceration was observed (Fig. 5d). The anti-apoptotic BCL-2 family profile did not change in the xenograft model (Supplementary Fig. 5A)

## Discussion

We demonstrated that high expression of MCL1 with low expression of BCL-X_L_ and BCL-2 was common in SCLC specimens from clinical patients, and S63845 was effective in high MCL1 with low BCL-X_L_ SCLC cell lines. Furthermore, based on our in vivo data, S63845 was as effective as CE; therefore, MCL1 inhibition therapy may improve the overall survival of SCLC patients.

MCL1 impedes BAK/BAX-dependent apoptosis, and S63845 inhibits the binding of BAK and BAX to MCL1, resulting in cancer cell death^24^. Single knockdown of BAK or BAX was insufficient to block S63845 efficacy. Co-IP assays supported that S63845 prevented BAK from binding to MCL1, as previously reported. However, MCL1 did not bind to BAX in our experiments. According to a previous study that reviewed the interactions among BCL-2 family members, MCL1 may not interact with BAX in some situations^39^. The binding affinity of MCL1 to BAX may not be strong in DMS114 and KTOR201. On the other hand, regardless of BAK expression, the interaction of MCL1 with BAK decreased according to the increase in S63845 concentration. This suggested that MCL1 inhibition therapy is dependent on the anti-apoptotic BCL-2 family members but not pro-apoptotic BCL-2 family members.

BCL-X_L_ is known as a resistance factor for MCL1 inhibition therapy. The sensitivity of SW1271 expressing both MCL1 and BCL-X_L_ to S63845 was restored by knockdown of BCL-X_L_. On the other hand, DMS53, which expresses MCL1, BCL-X_L_, and BCL-2, was slightly sensitized to S63845 despite BCL-X_L_ knockdown. As double knockdown of BCL-X_L_ and BCL-2 in DMS53 improved the sensitivity of S63845 more than single knockdown, BCL-2 may act as another resistance factor for S63845 sensitivity. In addition, TMA revealed that MCL1 expression was sometimes accompanied by high expression of BCL-X_L_ and BCL-2 or both; therefore, we selected navitoclax, which inhibits both BCL-X_L_ and BCL-2, to evaluate combination therapy. There are several therapeutic drugs that inhibit the anti-apoptotic BCL-2 family proteins. Several recent studies suggested targeting BCL-2 and BCL-X_L_ to be effective against SCLC^13,23^. However, the BCL-2/BCL-X_L_ inhibitor navitoclax did not have good tumor suppressive efficacy due to thrombocytopenia in a phase 2 trial^40^. As thrombocytopenia was caused by BCL-X_L_ inhibition, the BCL-2-specific inhibitor, venetoclax, is a good candidate to control SCLC. Indeed, it was effective against SCLC with high expression of BCL-2, whereas that with low expression of BCL-2 may have another anti-apoptosis mechanism^13^.

There are several studies on double inhibition of MCL1 and BCL-2. The combination of S63845 with venetoclax, which is a FDA-approved BCL-2 inhibitor, has potent activity against nasopharyngeal carcinoma, acute myeloid leukemia, and mantle cell lymphoma without severe toxicity^26,41,42^. Consistent with this, venetoclax and S63845 may have synergistic efficacy against SCLC. As our in vitro study suggested that BCL-X_L_ plays a role in reducing the efficacy of S63845, we focused on the combination of S63845 with navitoclax. Navitoclax was effective in the SCLC cell lines with high BCL-2 expression or high BCL-X_L_ with low MCL1 expression. The combination therapy was also effective in SCLC cell lines in the in vitro study. However, the therapeutic range of navitoclax with S63845 was narrow in in vivo studies. The comprehensive inhibition of MCL1, BCL-X_L_, and BCL-2 may be impractical in clinical settings.

Simultaneous evaluation of MCL1, BCL-X_L_, and BCL-2 has not been reported in SCLC tissue. In a previous report, the BCL-2 expression rate assessed by immunohistochemistry was 46.3% (*N* = 68)^43^, but that of MCL1 was not reported. The mRNA and protein expression levels did not agree with each other for MCL1, BCL-X_L_, and BCL-2. This may be due to changes in the half-lives of the proteins. S63845 extended the half-life of MCL1 without increasing MCL1 mRNA levels^24^. Therefore, it is important to evaluate protein expression regarding the anti-apoptotic BCL-2 family.

TMA immunohistochemistry demonstrated a MCL1^high^/BCL-X_L_^low^/BCL-2^low^ population in 48% of the examined cases. SCLC has been considered to consist of four major subtypes based on the expression of ASCL1, NEUROD1, YAP1, and POU2F3. The POU2F3 subtype was most observed in the MCL1^high^/BCL-X_L_^low^/BCL-2^low^ population and was not observed in the BCL-X_L_^high^ population. Although the relationship between POU2F3 and BCL-X_L_ remains to be fully studied, the POU2F3 subtype might be a candidate for MCL1 inhibition therapy.

Several limitations of our study need to be considered. First, it was not fully studied whether S63845 has off-target effects causing cell death. In principle, the knockdown of MCL1 does not completely abolish MCL1 function. A CRISPR/Cas9 knockout system would remove MCL1 function and help solve this problem. Second, the influence of other anti-apoptotic BCL-2 family members was not investigated in the study. If SCLC cell lines do not respond to MCL1 inhibition therapy, other anti-apoptotic BCL-2 family members should be studied. Third, comprehensive inhibition therapy was only considered with S63845 and navitoclax. Since both agents might have off-target effects, other combinations should be tried. Finally, regarding SCLC subtypes, NEUROD1 and POU2F3 antibodies might not be ideal for immunoblotting and it was difficult to define which subtype marker was dominantly positive in the TMA study, partially due to multiple positives or no positives. A larger TMA study will be necessary. In addition, the cut-off level for each immunohistochemical evaluation may not be applicable for clinical situations. Obtaining and analyzing both tissue samples and cell lines from the same patients would help solve the problem.

**Fig. 6:**
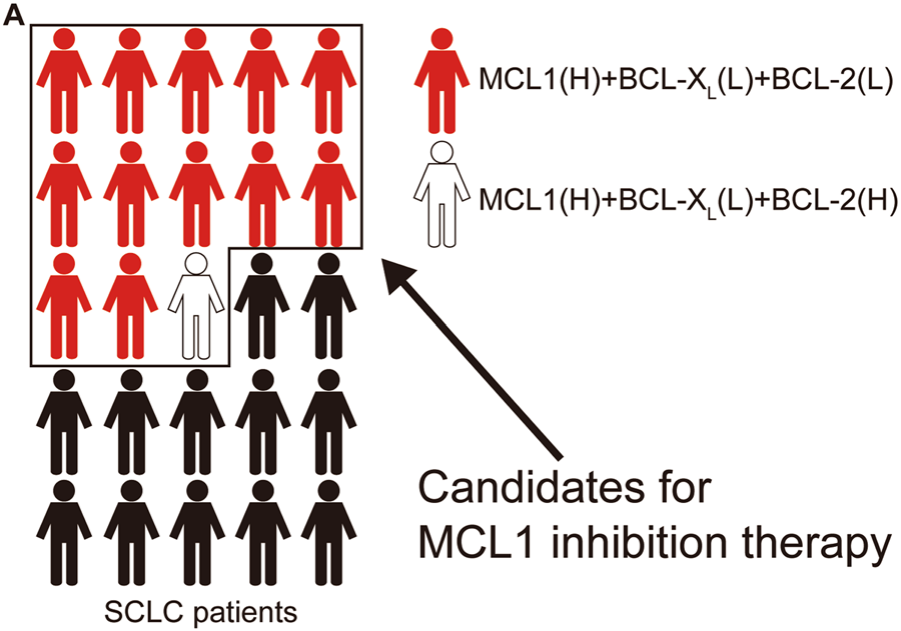
MCL1 targeting strategy model. **a** Schematic overview of the proposed strategy for MCL1 targeting therapy. (H) or (L) means high or low expression, respectively.

In conclusion, our study suggested that MCL1 inhibition is useful for the treatment of MCL1^high^/BCL-X_L_^low^ SCLC (Fig. 6). Once BCL-X_L_ and/or BCL-2 protein levels have increased, navitoclax may be the preferred treatment option. Several selective MCL1 inhibitors (AMG176, AMG397, and S64315) are currently in clinical trials for hematological malignancies^44^. Our study reinforces the importance of conducting clinical trials in SCLC patients while investigating the protein levels of MCL1, BCL-X_L_, and BCL-2.

## Supplementary information

Supplementary Figure Legends

Supplementary Figure 1

Supplementary Figure 2

Supplementary Figure 3

Supplementary Figure 4

Supplementary Figure 5

Supplementary Figure 6

Supplementary Figure 7

Supplementary Figure 8

Supplementary Tables
