# Supplementary material for: MCL1 inhibition is effective against a subset of small-cell lung cancer with high MCL1 and low BCL-XL expression
Source: Cell Death Dis. 2020 Mar 9;11(3):177. doi: 10.1038/s41419-020-2379-2 (PMC7063049; doi:10.1038/s41419-020-2379-2)
Supplement: Supplementary file 1 — Supplementary Figure Legends [file 41419_2020_2379_MOESM1_ESM.docx]

**Supplementary Figure Legends**

**Supplementary Figure 1.**

**A**, Representative cores showing ASCL1, NEUROD1, YAP1, and POU2F3 stainings in small-cell lung cancer samples from a tissue microarray.

**B**, Relative gene expression of *chromogranin A*, *synaptophysin*, and *N-CAM 180/140 kDa* normalized to *GAPDH* by qRT-PCR. Results are expressed as mean ± SEM (N = 4).

**Supplementary Figure 2.**

**A**, Caspase 8 and 9 activity in DMS114 and KTRO201 treated with 100 nM S63845 for 6 hours was significantly higher than that in SW1271 and DMS53. Data are presented as fold-change in activity relative to vehicle as means ± SEM (N = 9). One-way ANOVA with Tukey’s multiple comparison test was used. *, p < 0.05.

**B**, Caspase 3/7, 8, and 9 activity in DMS114 and KTRO201 treated with 200 nM S63845 for 6 hours was significantly higher than that in SW1271 and DMS53. Data are presented as fold-change in activity relative to vehicle as means ± SEM (N = 9). One-way ANOVA with Tukey’s multiple comparison test was used. *, p < 0.05.

**Supplementary Figure 3.**

**A**, Representative flow cytometry data to illustrate gating and quadrant strategy for FACS measurement of apoptosis. Flow cytometry density plots showing annexin V (X-axis) and propidium iodide (Y-axis) staining of SCLC cell lines 4 hour after treatment with DMSO or 100 nM S63845 in the presence or absence of 50 μM Z-VAD-FMK.

**Supplementary Figure 4.**

**A**, Cell viability assay of DMS114, KTOR201, DMS53, and SW1271 transfected with MCL1 siRNA or negative control siRNA, treated with S63845 72 hours after transfection. Results are expressed as mean ± SEM (N = 5). Two-way ANOVA with Tukey’s multiple comparison test demonstrated a significant difference between siNC and siMCL1 A (*) and between siNC and siMCL1 B (†), p < 0.05.

**B**, Cell viability assay of KTOR201 transfected with BCL-2 siRNA or negative control siRNA, treated with S63845 72 hours after transfection. Results are expressed as mean ± SEM (N = 5). Two-way ANOVA with Tukey’s multiple comparison test demonstrated no significant difference (n.s.) between siNC and siMCL1 A and between siNC and siMCL1 B, p < 0.05.

**C**, Immunoblotting analysis of lysates from KTOR201 following siRNA-mediated BCL-2 knockdown, which affected MCL1, BCL-X_L_, and BCL-2 expression.

**Supplementary Figure 5.**

**A**, Immunoblotting analysis of lysates from DMS114, KTOR201, and DMS53 of the xenograft tumors (Xeno).

**Supplementary Figure 6.**

**A**, Uncropped raw western blots of Figure 1E and Figure 1F.

**Supplementary Figure 7.**

**A**, Uncropped raw western blots of Figure 3A, Figure 3C, and Figure 3E.

**Supplementary Figure 8.**

**A**, Uncropped raw western blots of Figure 4B, Figure S4, and Figure S5.
