# Supplementary figures and images for: MCL1 inhibition is effective against a subset of small-cell lung cancer with high MCL1 and low BCL-XL expression
Source: Cell Death Dis. 2020 Mar 9;11(3):177. doi: 10.1038/s41419-020-2379-2 (PMC7063049; doi:10.1038/s41419-020-2379-2)

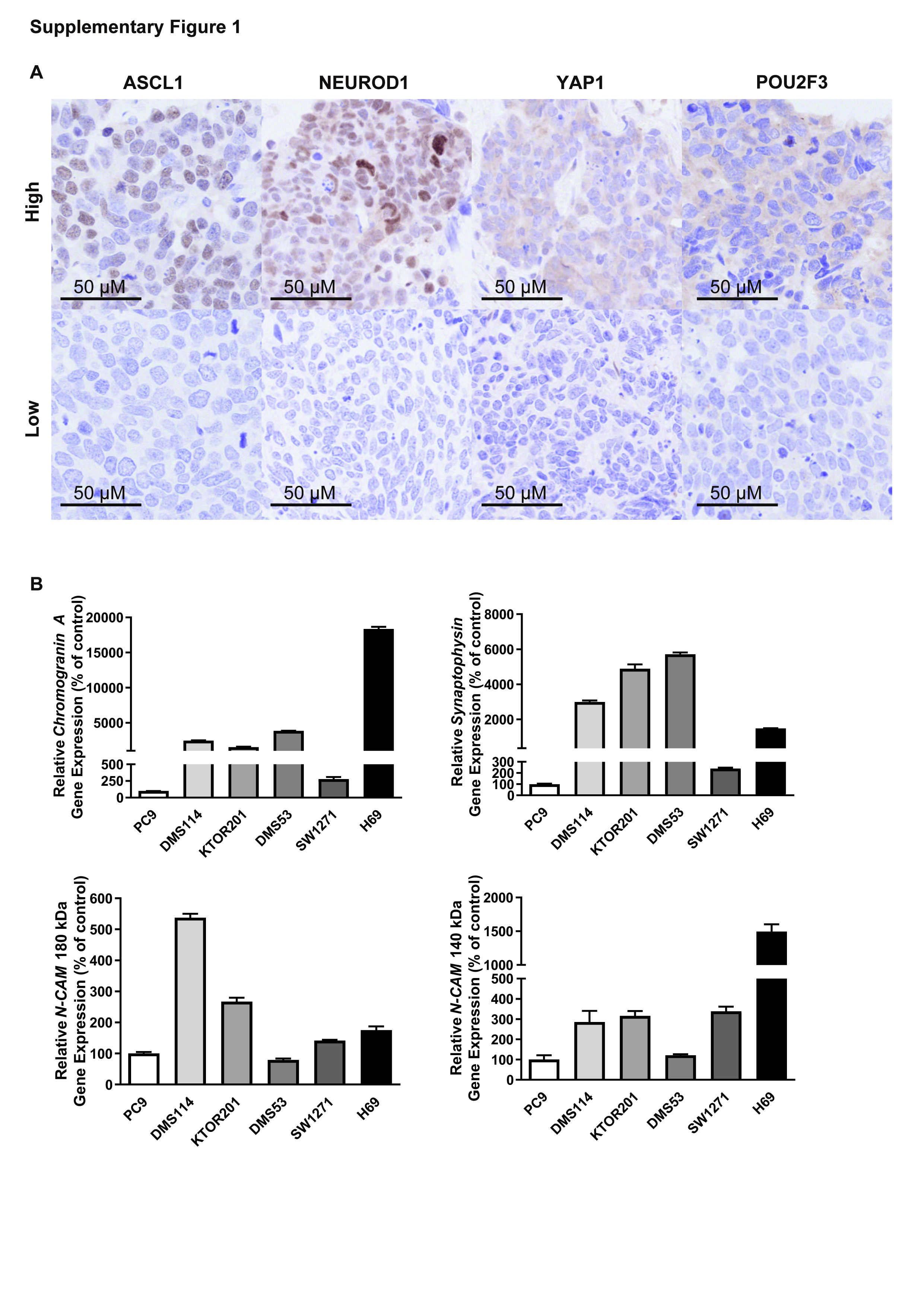

Supplement: Supplementary file 2 — Supplementary Figure 1 [file 41419_2020_2379_MOESM2_ESM.tif]

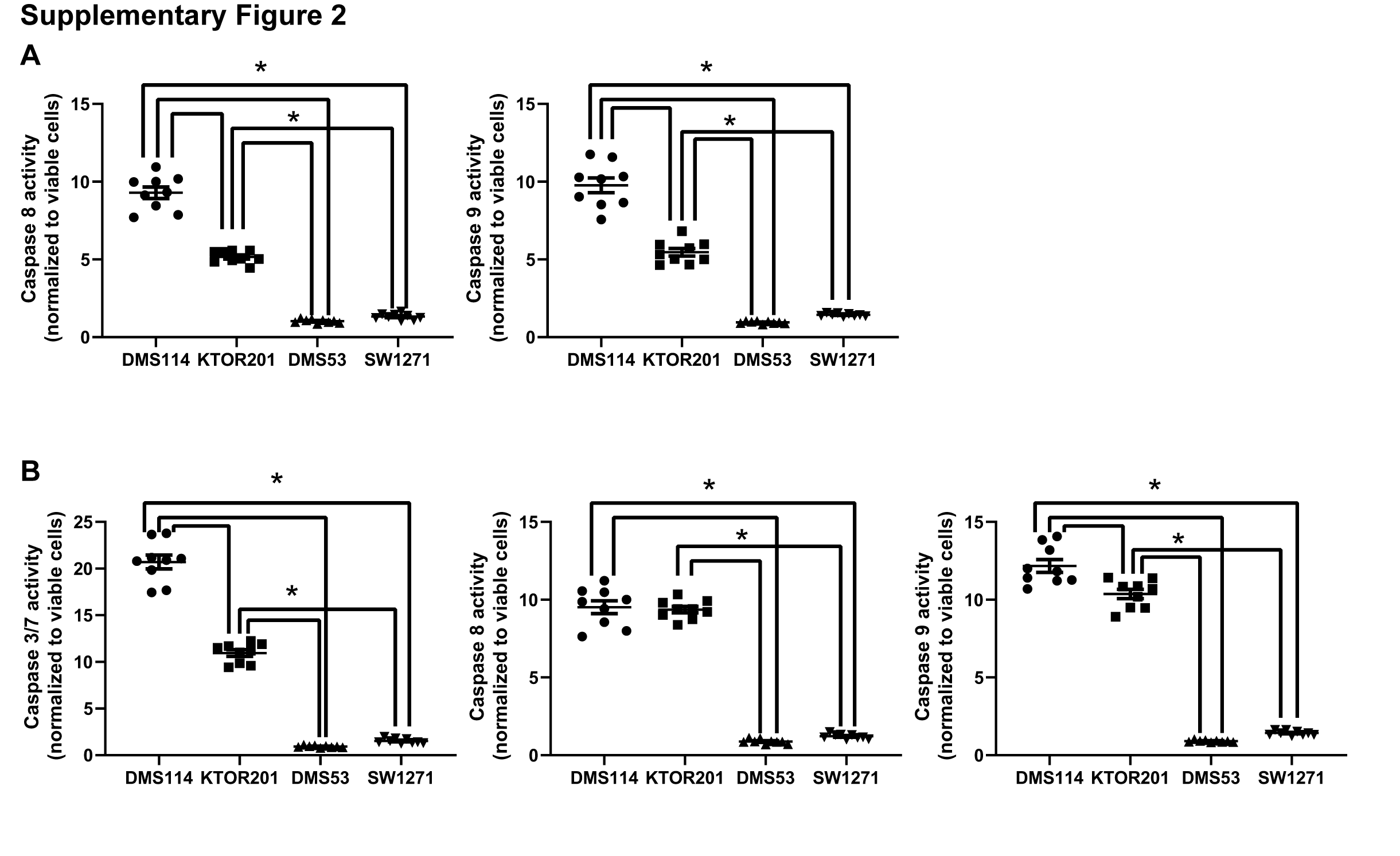

Supplement: Supplementary file 3 — Supplementary Figure 2 [file 41419_2020_2379_MOESM3_ESM.tif]

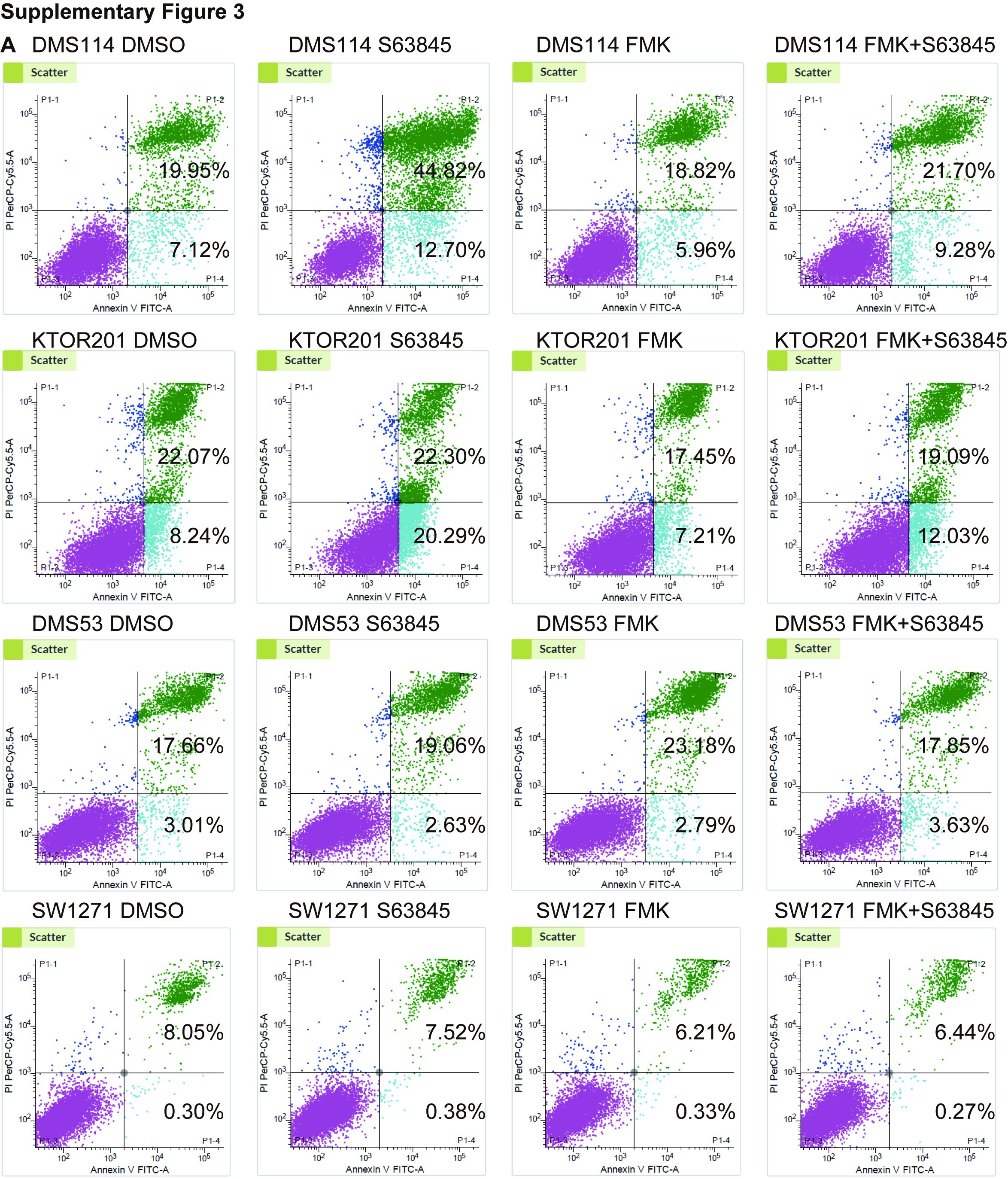

Supplement: Supplementary file 4 — Supplementary Figure 3 [file 41419_2020_2379_MOESM4_ESM.tif]

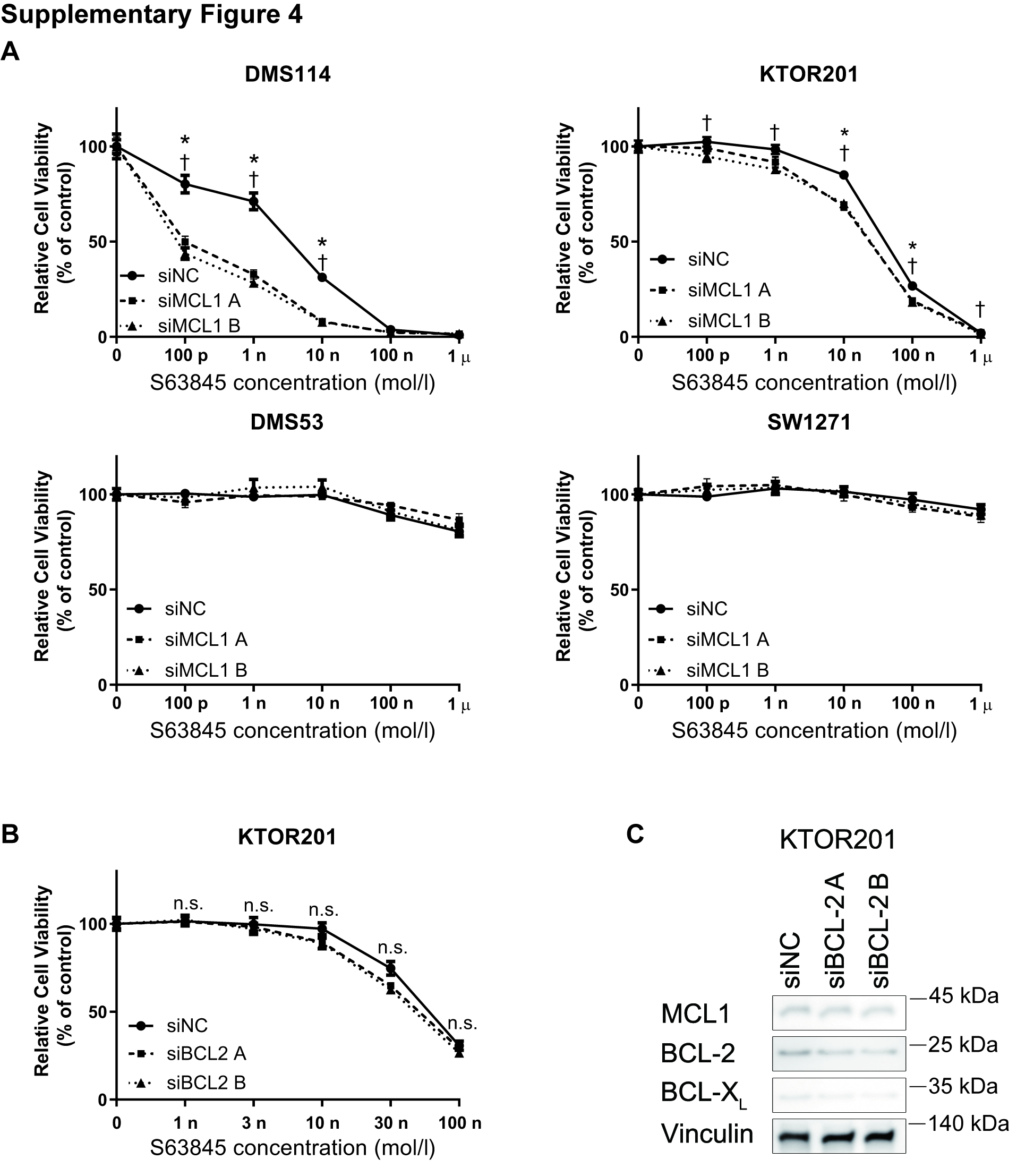

Supplement: Supplementary file 5 — Supplementary Figure 4 [file 41419_2020_2379_MOESM5_ESM.tif]

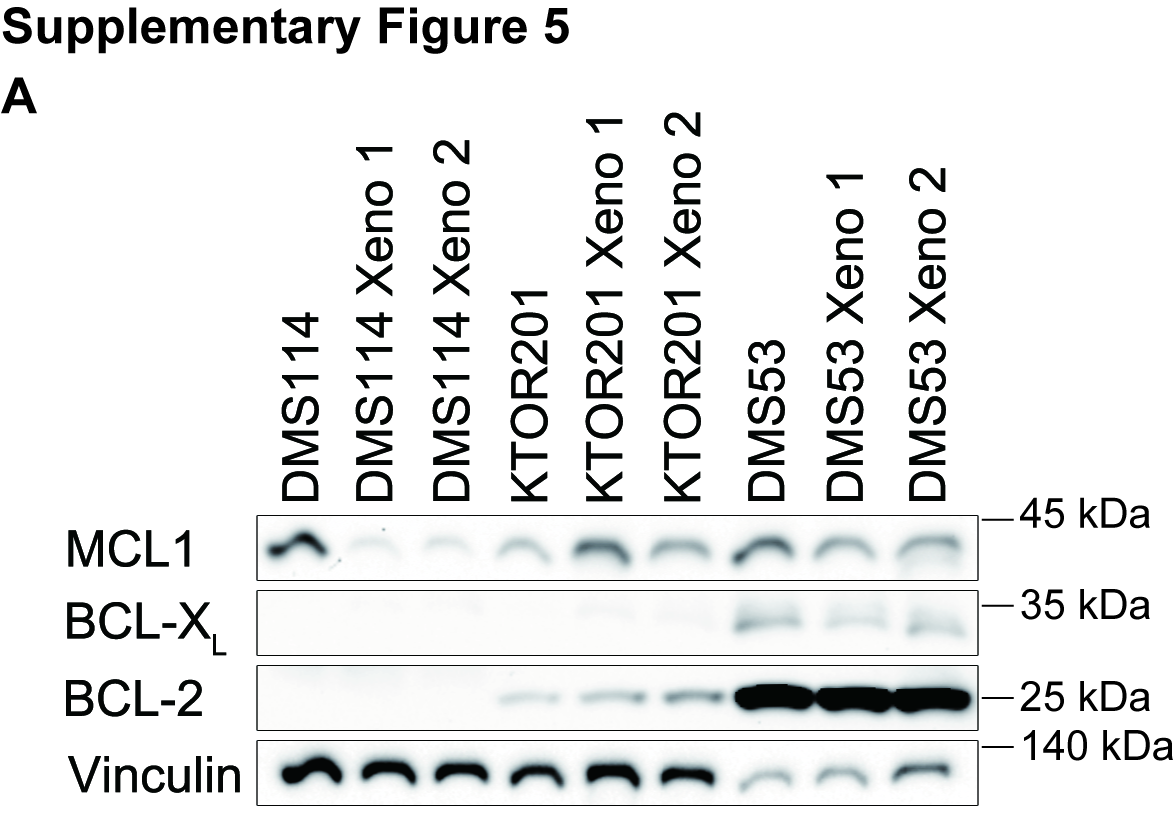

Supplement: Supplementary file 6 — Supplementary Figure 5 [file 41419_2020_2379_MOESM6_ESM.tif]

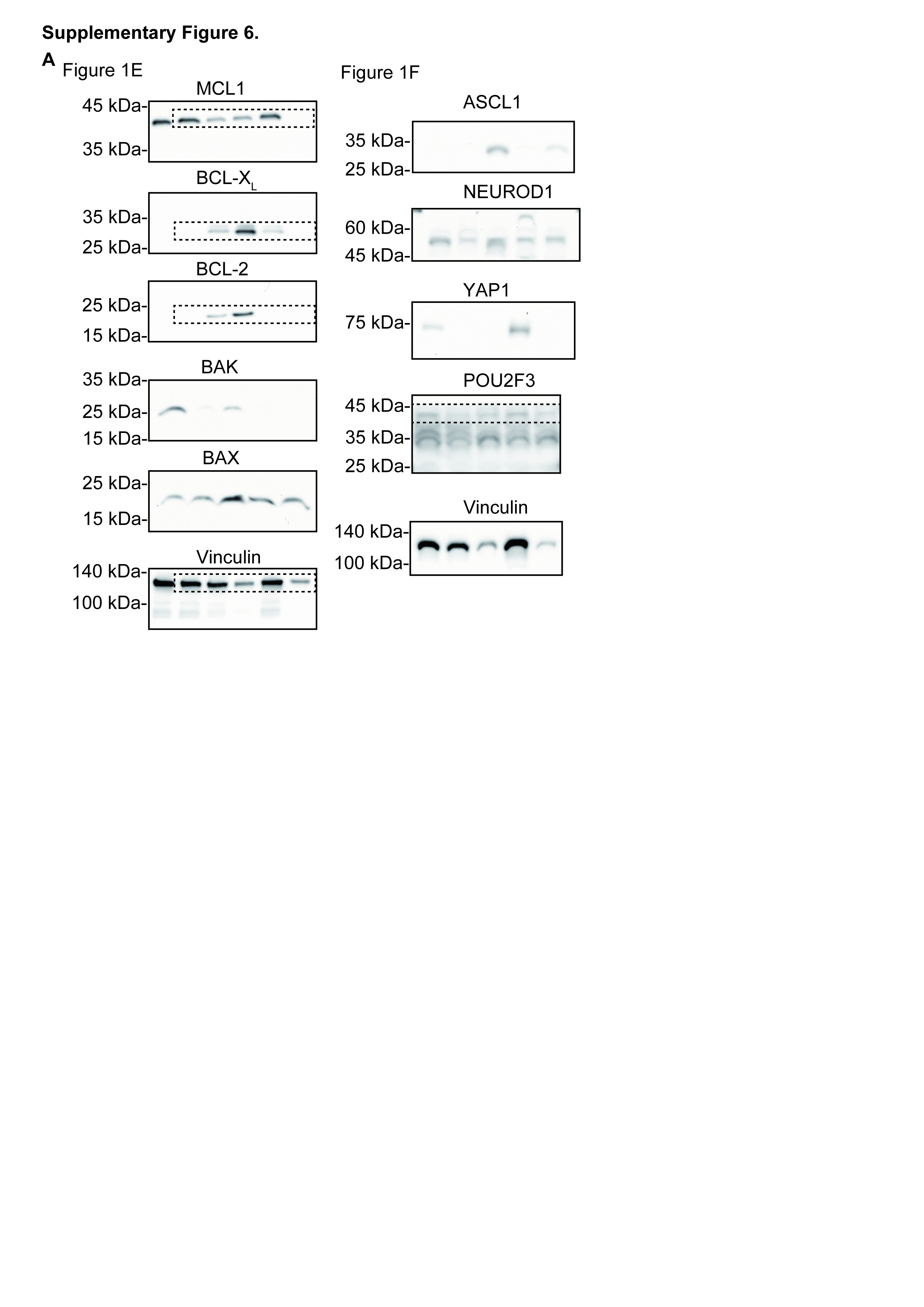

Supplement: Supplementary file 7 — Supplementary Figure 6 [file 41419_2020_2379_MOESM7_ESM.tif]

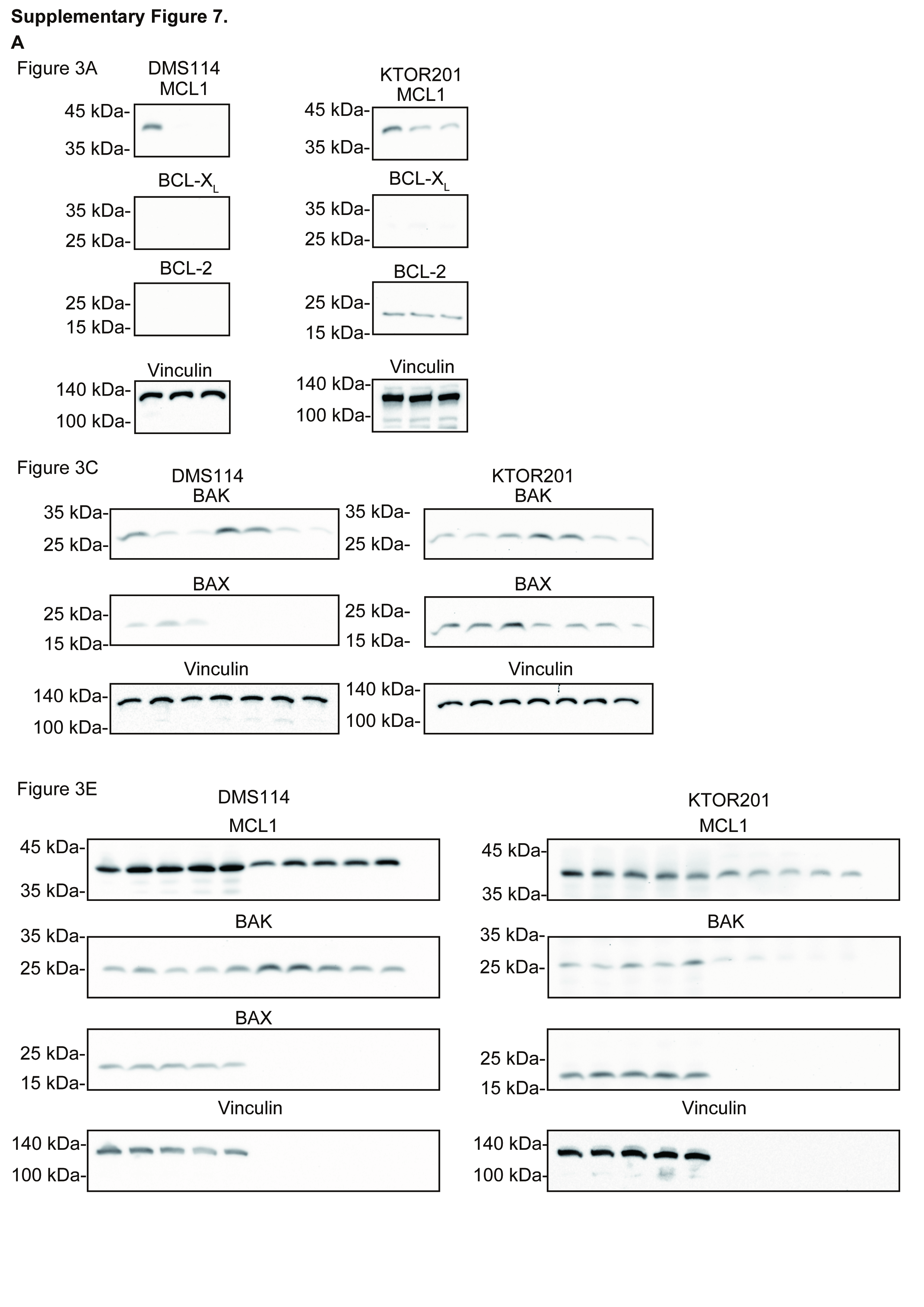

Supplement: Supplementary file 8 — Supplementary Figure 7 [file 41419_2020_2379_MOESM8_ESM.tif]

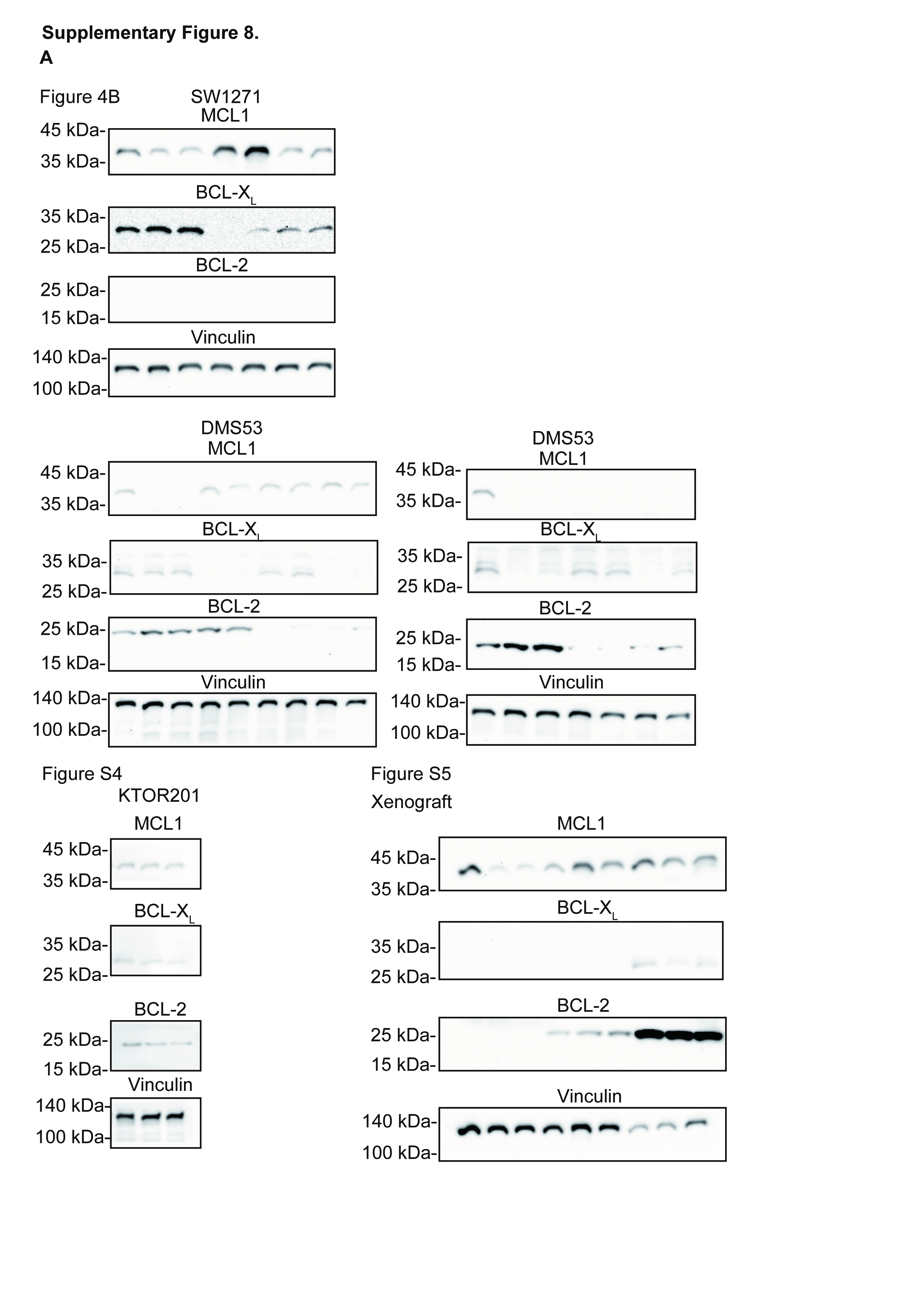

Supplement: Supplementary file 9 — Supplementary Figure 8 [file 41419_2020_2379_MOESM9_ESM.tif]
